# Association between Genetic Polymorphisms and Response to Anti-TNFs in Patients with Inflammatory Bowel Disease

**DOI:** 10.3390/ijms17020225

**Published:** 2016-02-06

**Authors:** Rocío Prieto-Pérez, Berta Almoguera, Teresa Cabaleiro, Hakon Hakonarson, Francisco Abad-Santos

**Affiliations:** 1 Clinical Pharmacology Service, Hospital Universitario de la Princesa, Instituto de Investigación Sanitaria la Princesa (IP), Madrid 28006, Spain; rociomaria.prieto@gmail.com (R.P.-P.); teresacabaleiro@hotmail.com (T.C.); 2 Instituto Teófilo Hernando, University Autónoma de Madrid (UAM), Madrid 28029, Spain; 3 Center for Applied Genomics, The Children’s Hospital of Philadelphia, Philadelphia, PA 19104, USA; balmoguera@hotmail.com (B.A.); hakonarson@email.chop.edu (H.H.); 4 Centro de Investigación Biomédica en Red de Enfermedades Hepáticas y Digestivas (CIBERehd), Instituto de Salud Carlos III, Madrid 28006, Spain

**Keywords:** inflammatory bowel disease, polymorphisms, pharmacogenomics, adalimumab, infliximab

## Abstract

Tumor necrosis factor (TNF) α is a major proinflammatory cytokine involved in the immune response in inflammatory bowel disease (IBD). Anti-TNF drugs such as infliximab and adalimumab are used to treat IBD; however, approximately 30% of patients do not respond to treatment. Individual genetic differences could contribute to lack of efficacy. Genetic studies have tried to uncover the factors underlying differences in response, however, knowledge remains limited, and the results obtained should be validated, so that pharmacogenetic information can be applied in clinical practice. In this review, we gather current knowledge in the pharmacogenetics of anti-TNF drugs in patients with IBD. We observed a connection between the major genes described as possible predictors of response to anti-TNF drugs in IBD and the cytokines and molecules involved in the T helper (Th) 17 pathway.

## 1. Introduction

Inflammatory bowel disease (IBD) comprises ulcerative colitis (UC) and Crohn’s disease (CD). Patients with IBD display some common symptoms such as severe diarrhea, pain, fatigue, and weight loss [1], but the localization is slightly different: whereas CD affects the whole gastrointestinal tract, UC primarily affects the distal intestine and ileum [2]. The prevalence of IBD varies with geographic location with higher rates for UC in Europe: 505/100,000 individuals *versus* 249/100,000 in North America and similar rates for CD 319-322/100,000 in both areas [3].

Genetic, environmental, and immunoregulatory factors play a key role in the development of IBD. Although its cause is unknown, IBD is characterized by a dysregulated response of the mucosal immune system to intraluminal bacterial antigens [4]. Specifically, the up-regulation of cytokines such as tumor necrosis factor (TNF) α, interleukin (IL) 1β, and IL6 [5,6], which activate T helper (Th) 1 and 17 cells have a central role in IBD [7].

Anti-TNF drugs are indicated and recommended in patients with moderate-to-severe IBD who do not tolerate or do not respond to conventional therapies. Infliximab and adalimumab are monoclonal antibodies that bind with high affinity to TNFα and block its interaction with cell surface receptors. Although both are effective in IBD [8,9], approximately 30% of patients do not respond to anti-TNF drugs (~30%) and may develop adverse reactions to the treatment [10,11,12]. It is increasingly being recognized that genetics may account for these inter-individual differences in the response to anti-TNF treatment [13]. Therefore, identification of genetic markers predictive of drug response, could help optimize treatments and prevent adverse reactions [14].

Based on current knowledge of pharmacogenetics in IBD, this review highlights the importance of Th17 cells and their relationship with the response to anti-TNF medication.

## 2. Immune System and Th17 Cells in IBD

**Figure 1:**
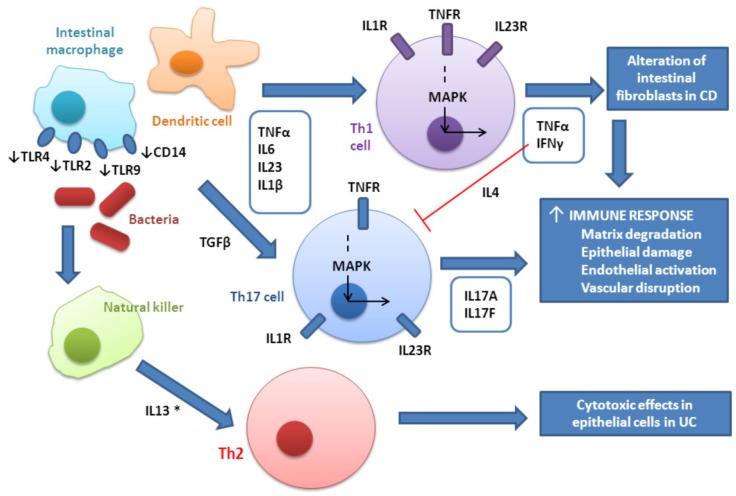
Immune response in inflammatory bowel disease (IBD). TLR: toll-like receptor; CD14: CD14 molecule; TNF: tumor necrosis factor; IL: interleukin; Th: lymphocyte T helper; IL1R: interleukin 1 receptor; TNFR: tumor necrosis factor receptor; IL23R: interleukin 23 receptor; IFN: interferon; MAPK: mitogen-activated protein kinase; CD: Crohn’s disease; UC: ulcerative colitis; ↑: upregulation; ↓: downregulation; *: regulation Th1 and Th17; →: stimulation; ⊥: inhibition.

IBD is characterized by excessive and abnormal immune response against commensal flora in genetically susceptible individuals, which involves both innate and adaptive immunity [4]. Adaptive immunity includes immunoglobulins produced by B cells and a mixture of Th1 cells, which are the predominant type in CD, and Th2, primarily observed in UC [15]. Th17, a CD4 T-cell lineage distinct from Th1 and Th2, which is promoted by IL23 and characterized by the production of IL17, has also been observed in IBD [16]. A schematic representation of the interconnection between the three Th cytokine profiles in IBD is illustrated in Figure 1.

The presence of microbes triggers Th1 development and the production of IL-12 and interferon γ (IFNγ), which then activate macrophages. Macrophage and dendritic cells produce the pro-inflammatory cytokines TNFα, IL6, IL23, and IL1β that promote differentiation of naïve CD4+ T cells into Th1 and Th17 [17]. Other cytokines such as Toll-like receptor (TLR) 3, TLR4, TLR9, and transforming growth factor β (TGFβ) are also involved in Th17 differentiation [18,19,20]. Smythies *et al.* reported that bacterial recognition receptors (TLR and CD14) are downregulated in resident intestinal macrophages. This allows these types of cells to reside in the distal ileum and colon, where the bacterial concentration is high [21]. Although TLRs are essential for the recognition of pathogens and activation of innate immunity, different types of TLRs bind to different molecules. For example, TLR4 binds to bacterial lipopolysaccharide, TLR2 binds to peptidoglycan [7], and TLR9 binds to various bacterial DNA species [22]. Polymorphisms in these receptors could influence the response to anti-TNF therapy in patients with IBD through alteration of the NFκβ pathway [23].

Activated Th1 cells produce IFNγ and TNFα. IFNγ inhibits differentiation to Th17, which is suggested to enhance the development of pathogenic Th17 cells and exacerbate autoimmunity [24] and also stimulates macrophage and dendritic cells, increasing the production of pro-inflammatory cytokines and, therefore, the immune response [6].

The Th2 response observed in UC seems to be an atypical cytotoxic response [7] mediated by non-classic natural killer T cells (activated by antigen-presenting cells) that produce IL13 [25]. Although the role of IL13 is not clear, variations in the *IL13* gene result in deregulation of the Th1 and Th17 pathways in related autoimmune diseases such as psoriatic arthritis [26].

Th17-cell development is driven by TGFβ and IL-6, whereas IL-23 seems to expand and maintain Th17-cell populations. Th17 cells produce several cytokines, such as IL17A and IL17F [27], with promotes the expression of TNFα. TNFα acts on intestinal fibroblasts, leading to the release of other cytokines (IL13 or TGFβ) and formation of strictures and fibrosis in CD [28]. Moreover, TNFα and the proinflammatory cytokines IL6 and IL1 produce matrix degradation, epithelial damage, endothelial activation, and vascular disruption in CD (Figure 1) [6].

## 3. Polymorphisms Associated with Response to Anti-TNF Drugs in IBD

There are currently three TNFα antagonists available in the treatment of IBD: infliximab, adalimumab and golimumab, which are full-length antibodies and their Fc region is capable of complement fixation and Fc-receptor mediated biologic activities. These compounds exert a down-regulation of inflammatory cells in the inflamed bowel mucosa induced by apoptosis in TNF carrying cells [29].

Anti-TNF drugs are safe and effective for treatment of IBD [8,30,31] but close to 30% of patients are non-responders [23,30]. Genetics and immune system play an important role in the development of IBD, and differences in response could be due to the patient’s genetic background [32]. However, knowledge of the pharmacogenetics of anti-TNF therapy in IBD is limited, and more studies are necessary before personalized medication can be applied to clinical practice. Such approaches may be able to predict which patients with IBD would be more likely to respond to anti-TNF drugs in order to minimize the risks for the patients and the development of adverse reactions.

Table 1 presents the most updated information on the pharmacogenetics of IBD with respect to anti-TNF drugs (infliximab and/or adalimumab).

In a study of luminal CD (*n* = 204) receiving infliximab, patients with the TT genotype for rs4645983 (*CASP9*) or CC/CT genotype for rs763110 (*FASLG*) showed a better response to the treatment at week 4 [30]. Hlavaty *et al.* also reported that concomitant therapy of infliximab with azathioprine/mercaptopurine could improve the effect of unfavorable genotypes [30]. Another variant in *FASLG*, rs763110, was able to predict the therapeutic response to infliximab in a sample of patients with fistulizing CD (*n* = 83) at week 10 [30]. Similarly, Japanese patients with CD and the GG genotype for *FCGR3A* had a better response at week 8 [33].

*ATG16L1* was recently postulated as a predictor of therapeutic response for adalimumab. *ATG16L1* is an autophagy-related gene that processes intracellular bacteria [34] and is expressed in intestinal epithelial cell lines [35]. *ATG16L1* was associated with higher susceptibility to CD [34,35], and patients with the TT genotype for rs10210302 responded better to adalimumab after 12, 20, and 30 weeks of treatment compared to the CC genotype [36].

The cytokine IL23 is involved in the pathogenesis of IBD (Figure 1). The single-nucleotide polymorphism (SNP) rs11209026 in the *IL23R* gene, which encodes a subunit of the receptor for IL23, has been associated with CD [37] and Duerr *et al.* suggested that IL23R could be a therapeutic target in IBD [37]. Moreover, several genetic variants in *IL23R* have been associated with response to infliximab in patients with moderate-to-severe UC (*n* = 90) at week 14 (Table 1) [31]. For instance, AA genotype for rs1004819, rs10889677, and rs11209032, GG genotype for rs2201841, and CC genotype for rs1495965 in *IL23R* gene increased the probability to respond to infliximab [31]. However, GG genotype for rs7517847 and rs11465804, CC genotype for rs10489629, and AA genotype for rs1343151 in *IL23R* decreased the probability to respond to this drug [31]. Therefore, IL23R could be an interesting molecule for further follow-up.

IL23 is released with other relevant pro-inflammatory cytokines like IL6, TNFα, and IL1β during the immune response in IBD (Figure 1). In a recent study, Bank *et al.* found the TC/CC genotype for rs10499563 in *IL6* and the GA/AA genotype with a better response to anti-TNF. The authors investigated the role of three genetic variants in *IL1β*, for rs4848306, rs1143623 and rs1143627, but only found a positive association for rs4848306 [23]. Bank and colleagues also studied the effect of rs4251961 in *IL1RN*, which regulates IL1β signaling in immune and inflammatory responses, and observed that allele C was associated with poorer responses to therapy [23].

In contrast to Bank’s findings on *IL1β*, Lacruz-Guzmán and colleagues found a poorer response to infliximab in carriers of the C allele of rs1143634, in CD patients at 14 weeks [38]. These authors also evaluated the association between several polymorphisms in *TNFα* (rs361525, rs1800629, rs1799724) and response to infliximab but did not find any significant association [38], as opposed to what has been reported by several other authors (Table 1) [23,39,40].

Taylor *et al.* described an association between homozygosity for the NcoI-TNFc-aa13L-aa26 haplotype (1-1-1-1) in the *LTA* gene, which encodes a member of the TNF family, the lymphotoxin-α precursor, and poor response to infliximab [41]. Other TNF family members such as the receptors 1A and 1B have been associated with response to this compound (Table 1) [23,32]: rs767455 in *TNFRSF1A* and rs1061622, rs1061624, and rs3397 in *TNFRSF1B* [32] (Table 1).

A polymorphism in *TNFAIP3*, rs6927172 was reported by Banks *et al.* associated with poor response to anti-TNF therapy [23]. *TNFAIP3* encodes the α-induced protein, whose expression is induced by TNFα, and that inhibits NFκβ activation (reference). Banks also investigated other components of the NFκβ pathway and found no association with polymorphisms in the *NFKBIA* and *NFKB1* genes but a positive association between rs7222094 in *MAP3K14* and anti-TNF medication response [23].

*TLR9*, *TLR2*, and *TLR4* involved in the recognition of pathogens and activation of the immune response (Figure 1), have also been associated with response to anti-TNF therapy in IBD (Table 1) [23]. TLR4 cooperates with LY96 and CD14 to mediate the innate immune response to bacterial lipopolysaccharide. The A allele of rs2569190 in *CD14* and the G allele of rs11465996 in *LY96* have been associated with anti-TNF response (Table 1) [23]. However, rs5744168 in *TLR5* and rs12377632 in *TLR4* did not show any association with response [23]. A study on patients with CD with adalimumab, focused on genetic variants in *CD14* and *TLR4* was not able to replicate the above findings [42].

Fujino *et al.* found mRNA expression and serum levels of IL17 to be increased in patients with IBD [43] and suggested that IL17 might be associated with altered immune and inflammatory responses in the intestinal mucosa (Figure 1). This cytokine seems to play a relevant role in the response to anti-TNF drugs in patients with IBD (Table 1). Patients with this disease and A allele carriers for rs2275913 in *IL17A* had a poorer response to treatment with anti-TNF drugs [23].

Finally, INFγ is also produced by Th1 and regulates Th17 cells (Figure 1). The SNP rs2430561 in this cytokine seems to influence the response to anti-TNF therapy in patients with IBD [23].

**Table 1:** Association between single-nucleotide polymorphisms SNPs and response to anti-tumor necrosis factor (TNF) drugs (infliximab and/or adalimumab) in patients with inflammatory bowel disease.

Gene	SNP	CHR	Minor Allele	MAF	Sample Size Studied	Population	Effect on the Response	Disease/Drug	Involved in Th17 Responses	Reference
*CASP9*	rs4645983	1	A	0.218	287	Caucasian	↓ C allele	CD/I	NO	[30]
*FASLG*	rs763110	1	C	0.607	287	Caucasian	↑ C allele	CD/I	NO	[30]
*FCGR3A*	rs396991	1	T	-	102	Japanese	↑ GG genotype	CD/I	NO	[33]
*IL23R*	rs1004819	1	A	0.288	90	Caucasian	↑ AA genotype	UC/I	YES	[31]
*IL23R*	rs2201841	1	G	0.296	90	Caucasian	↑ GG genotype	UC/I	YES	[31]
*IL23R*	rs10889677	1	A	0.292	90	Caucasian	↑ AA genotype	UC/I	YES	[31]
*IL23R*	rs11209032	1	A	0.306	90	Caucasian	↑ AA genotype	UC/I	YES	[31]
*IL23R*	rs1495965	1	C	0.410	90	Caucasian	↑ CC genotype	UC/I	YES	[31]
*IL23R*	rs7517847	1	G	0.442	90	Caucasian	↓ GG genotype	UC/I	YES	[31]
*IL23R*	rs10489629	1	C	0.473	90	Caucasian	↓ CC genotype	UC/I	YES	[31]
*IL23R*	rs11465804	1	G	0.045	90	Caucasian	↓ GG genotype	UC/I	YES	[31]
*IL23R*	rs1343151	1	A	0.327	90	Caucasian	↓ AA genotype	UC/I	YES	[31]
*TNFRSF1B*	rs1061622	1	G	0.239	81	Japanese	↓ G allele	CD/I	YES	[32]
*TNFRSF1B*	rs1061624	1	G	0.477	81	Japanese	#	CD/I	YES	[32]
*TNFRSF1B*	rs3397	1	T	0.526	81	Japanese	#	CD/I	YES	[32]
*ATG16L1*	rs10210302	2	T	0.392	102	Caucasian	↑ T allele	CD/A	NO	[36]
*IL1B*	rs4848306	2	A	0.464	738	Caucasian	↑ A allele	CD, UC/A, I	YES	[23]
*IL1B*	rs1143634	2	A	0.208	47	Caucasian	↓ C allele	CD/I	YES	[38]
*IL1RN*	rs4251961	2	C	0.385	738	Caucasian	↓ C allele	CD, UC/A, I	YES	[23]
*TLR9*	rs187084	3	G	0.341	738	Caucasian	↑ TC genotype	CD, UC/A, I	YES	[23]
*TLR9*	rs352139	3	T	0.518	738	Caucasian	↓ AA gentoype	CD, UC/A, I	YES	[23]
*TLR2*	rs4696480	4	A	0.000	738	Caucasian	↓ TT genotype	CD, UC/A, I	YES	[23]
*TLR2*	rs11938228	4	A	0.296	738	Caucasian	↓ A allele	CD, UC/A, I	YES	[23]
*TLR2*	rs1816702	4	T	0.138	738	Caucasian	↑ T allele	CD, UC/A, I	YES	[23]
*TLR2*	rs3804099	4	C	0.451	738	Caucasian	↑ C allele	CD, UC/A, I	YES	[23]
*CD14*	rs2569190	5	A	0.474	738	Caucasian	↓ A allele	CD, UC/A, I	YES	[23]
*IL17A*	rs2275913	6	A	0.354	738	Caucasian	↓ A allele	CD, UC/A, I	YES	[23]
*LTA*	rs909253	6	G	0.358	59	Caucasian	##	CD/A, I	YES	[41]
*TNF*	rs361525	6	A	0.061	738, 82	Caucasian	↓ GA genotype	CD, UC/A, I	YES	[23,39]
*TNF*	rs1800629	6	A	0.173	82	Caucasian	↓ A allele	CD, UC/A, I	YES	[39]
*TNF*	rs1799724	6	T	0.099	98	Japanese	↓ C allele	CD/I	YES	[40]
*TNFAIP3*	rs6927172	6	G	0.175	738	Caucasian	↓ G allele	CD, UC/A, I	YES	[23]
*IL6*	rs10499563	7	C	0.195	738	Caucasian	↑ C allele	CD, UC/A, I	YES	[23]
*LY96*	rs11465996	8	G	0.358	738	Caucasian	↑ G allele	CD, UC/A, I	YES	[23]
*TLR4*	rs5030728	9	A	0.305	738	Caucasian	↑ A allele	CD, UC/A, I	YES	[23]
*TLR4*	rs1554973	9	C	0.217	738	Caucasian	↓ C allele	CD, UC/A, I	YES	[23]
*IFNG*	rs2430561	12	A	0.280	738	Caucasian	↑ A allele	CD, UC/A, I	YES	[23]
*TNFRSF1A*	rs4149570	12	A	0.336	738	Caucasian	↑ TT genotype	CD, UC/A, I	YES	[23]
*TNFRSF1A*	rs767455	12	C	0.508	738	Caucasian	↓ T allele	CD/A, I	YES	[32]
*MAP3K14*	rs7222094	17	T	0.381	738	Caucasian	↑ TC gentoype	CD, UC/A, I	YES	[23]

SNP: single-nucleotide polymorphism; CHR: chromosome; MAF: minor allele frequency in Caucasian population (information obtained from: HapMap (Available online: http://hapmap.ncbi.nlm.nih.gov/) or NCBI (Available online: http://www.ncbi.nlm.nih.gov/snp) web page); CD: Crohn’s disease; UC: ulcerative colitis; ↑: better response to anti-TNFs; ↓: poorer response to anti-TNFs; I: infliximab; A: adalimumab; *FASLG*: the apoptosis inducing ligand or Fas ligand; *CASP9*: caspase 9; *FCGR3A*: Fc γ receptor; IL23R: interleukin 23 receptor; *TNFRSF1B*: tumor necrosis factor (TNF) receptor superfamily 1B; *ATG16L1*: autophagy related 16-like 1; *IL1B*: interleukin 1 B; *IL1RN*: interleukin 1 receptor antagonist; *TLR*: toll-like receptor; *CD14*: CD14 molecule; *IL17A*: interleukin 17 A; *LTA*: lymphotoxin α; *TNFAIP3*: TNFα-induced protein 3; *IL6*: interleukin 6; *LY96*: lymphocyte antigen 96; *IFNG*: interferon γ; *TNFRSF1A*: TNF receptor superfamily 1A; *MAP3K14*: mitogen-activated protein kinase kinase kinase 14; # Significant results for AT haplotype (rs1061624 and rs3397, respectively); ## Homozygotes for the LTA NcoI-TNFc-aa13L-aa26 haplotype 1-1-1-1.

## 4. Conclusions

In conclusion, most of the genes associated with response to anti-TNF drugs in patients with IBD are associated with the Th17 pathway. However, data are limited, and further research is necessary to increase our knowledge of the Th17 process and understand its implication in response to anti-TNF drugs.
